# Does laser diode irradiation improve the degree of conversion of simplified dentin bonding systems?

**DOI:** 10.1590/1678-7757-2016-0461

**Published:** 2017

**Authors:** Leticia Ferreira de Freitas BRIANEZZI, Rafael Massunari MAENOSONO, Odair BIM, Giovanna Speranza ZABEU, Regina Guenka PALMA-DIBB, Sérgio Kiyoshi ISHIKIRIAMA

**Affiliations:** 1 Universidade de São Paulo, Faculdade de Odontologia de Bauru, Departamento de Dentística, Endodontia e Materiais Odontológicos, Bauru, SP, Brasil.; 2 Universidade de São Paulo, Faculdade de Odontologia de Ribeirão Preto, Departamento de Odontologia Restauradora, Ribeirão Preto, SP, Brasil.

**Keywords:** Dentin-bonding agents, Lasers, Physical properties

## Abstract

Simplified dentin-bonding systems are clinically employed for most adhesive procedures, and they are prone to hydrolytic degradation.

**Objective:**

This study aimed to investigate the effect of laser diode irradiation on the degree of conversion (DC), water sorption (WS), and water solubility (WSB) of these bonding systems in an attempt to improve their physico-mechanical resistance.

**Material and Methods:**

Two bonding agents were tested: a two-step total-etch system [Adper™ Single Bond 2, 3M ESPE (SB)] and a universal system [Adper™ Single Bond Universal, 3M ESPE (SU)]. Square-shaped specimens were prepared and assigned into 4 groups (n=5): SB and SU (control groups – no laser irradiation) and SB-L and SU-L [SB and SU laser (L) – irradiated groups]. DC was assessed using Fourier transform infrared spectroscopy with attenuated total reflectance. Additional uncured resin samples (≈3.0 µL, n=5) of each adhesive were also scanned for final DC calculation. For WS/WSB tests, similar specimens (n=10) were prepared and measured by monitoring the mass changes after dehydration/water storage cycles. For both tests, adhesive fluids were dropped into standardized Teflon molds (6.0×6.0×1.0 mm), irradiated with a 970-nm laser diode, and then polymerized with an LED-curing unit (1 W/cm^2^).

**Results:**

Laser irradiation immediately before photopolymerization increased the DC (%) of the tested adhesives: SB-L>SB>SU-L>SU. For WS/WSB (μg/mm^3^), only the dentin bonding system (DBS) was a significant factor (p<0.05): SB>SU.

**Conclusion:**

Irradiation with a laser diode improved the degree of conversion of all tested simplified dentin bonding systems, with no impact on water sorption and solubility.

## Introduction

Previous studies have indicated that an increase in temperature could enhance the mechanical properties of dentin bonding systems^7,25^. Despite these advantages, some concerns limit their clinical indications, since the heat could damage pulp tissue, thereby compromising dental vitality^14,27^.

In this scenario, the association of lasers with dentin bonding systems has been investigated to achieve a more resistant hybrid layer. Gonçalves, et al.^13^ (1999) assessed Nd:YLF laser irradiation over a three-step, etch-and-rinse system prior to curing, which promoted an increase in dentin bond strength values. These authors attributed this performance to the creation of a new substrate composed of recrystallized hydroxyapatite after being melted in the presence of resin monomers, resulting in a substrate that is physically more resistant. With the same purpose, Maenosono, et al.^17^ (2015) also showed that the use of a laser diode improved bond strength when associated with simplified dentin bonding systems (SDBSs). In addition to the role of the laser’s interaction with dentin, the authors also emphasized the evaporation of solvents as an advantage of laser use, reducing the bond’s susceptibility to degradation over time^16^.

Both lasers presented similar wavelengths (1047 nm for Nd:YLF, and 970 nm for laser diode), which partially explains the successful performance in these studies. As the laser diode presents additional interesting characteristics, such as versatility, smaller dimensions, and lower cost, it appears to be the more attractive option^17^.

Despite these favorable performances by bond-strength tests, it is important to understand how lasers affect the polymerization process of SDBSs. Any strategies that could reduce their susceptibility to hydrolytic degradation are desirable, as most of their failure is attributed to this limitation^24^. Water is an essential component for the hybridization process, as it produces expansion of the collagen fibrils, thereby allowing the penetration of dental adhesives into demineralized dentin^21,23^. However, residual water in the hybrid layer leads to hydrolytic degradation, impairing the polymerization of the dental adhesives and increasing their solubilization^3^.

Therefore, this study aimed to analyze the influence of laser diode irradiation on the degree of conversion (DC) and water sorption/solubility (WS/WSB) of uncured SDBSs. The null hypotheses were as follows: (1) there is no difference in the DC of SDBSs irradiated or not with laser diode and (2) there is no difference in the WS/WSB of SDBSs irradiated or not with laser diode.

## Material and methods

### Experimental design

For DC and WS/WSB, this study involved two factors: a laser at two levels (irradiated or not with laser diode) and the simplified dentin bonding system at two levels [Adper™ Single Bond 2 (3M ESPE, St Paul, Minnesota, USA) (SB) and Adper™ Single Bond Universal (3M ESPE, St Paul, Minnesota, USA) (SU)]. The quantitative response variables were DC (%), WS (μg/mm3), and WSB (μg/mm3).

**Figure 1:**
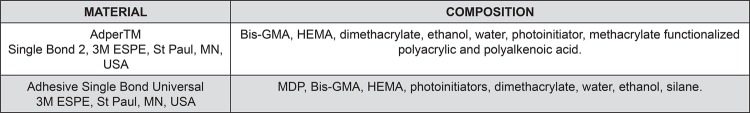
Chemical composition of the adhesive systems used according to the manufacturers

The materials used are described in Figure 1.

Bis-GMA=Bisphenol A and glycidyl methacrylate; HEMA=2-hydroxyethyl methacrylate; MDP= 10-Methacryloyloxydecyl dihydrogen phosphate

### Sample preparation

This study was performed in line with ISO 4049:2000 standard specifications, except for the specimen dimensions. Square-shaped Teflon molds (6.0×6.0×1.0 mm) were used to prepare the samples. The SDBSs were dropped to fill them. The specimens were air-dried smoothly for 20 s, from a distance of 10 cm, to help solvent evaporation^6,9,15^.

**Figure 2:**
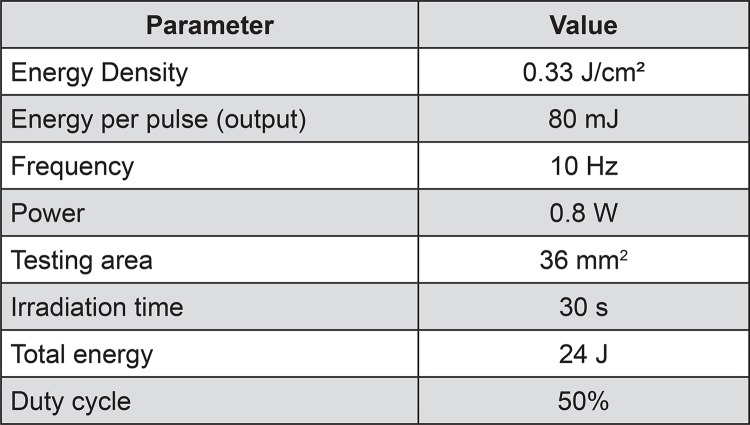
Laser diode parameters used for irradiation of the testing areas

In the laser groups (L), the SDBSs were irradiated with a laser diode (Siro LASER, Sirona Dental Systems, Benshein, Hessen, Germany) with an energy density of 0.33 J/cm^2^. The fiber tip was positioned toward the contact mode in the center of the adhesive at an inclination of 90° for automatic zigzag scanning (BioPDI XY Table, São Carlos, SP, Brazil) in the predetermined area. The scanning time was set at 30 s, and the offset in the y-axis was based on the thickness of the optical fiber tip (200 µm). The parameters used for laser diode irradiation are described in Figure 2 ^17^.

During the sequence, air bubbles were eliminated from the surface, and a polyester strip was placed over the adhesive, which was then covered with a glass slide to avoid contact of the fluid adhesive with oxygen during polymerization^15^. Then, the SDBSs were cured with an LED Blue Star 2 light (Microdont, São Paulo, SP, Brazil) at a power density of 1000 mW/cm^2^ for 20 s. Care was taken to place the tip perpendicularly to the sample surface, covering the entire specimen surface.

### DC

In general, when attenuated total reflectance (ATR)-Fourier transform infrared spectroscopy (FTIR) is used to calculate the DC, each SDBS is commonly dropped on the ATR crystal, and one run is performed. Subsequently, the same sample is polymerized, and the measure is taken again. However, it was necessary to standardize laser irradiation in this study, which implied the need for two different specimens for each condition. Additionally, square-shaped Teflon molds were used to prepare the specimens.

To calculate the DC, it was necessary to use the mean absorbance measured after curing and before curing, thus obtaining a single value for the uncured sample.

### DC test

An FTIR spectrometer (Shimadzu Corporation, Model IR Prestige 21, Kyoto, Honshu, Japan) was used with ATR (Smart Miracle^TM^ with diamond plate, Pike Technologies, Madison, Wisconsin, USA). Uncured resin samples (≈3.0 µL, n=5) of each adhesive were scanned, and the data were collected. Subsequently, new specimens were cured and stored for 24 hours in Eppendorf flasks at 37°C until analysis. Before the readings, they were compressed against the ATR crystal with a micrometric low-pressure clamp (408 psi) to allow optimal sample contact with it. The absorption spectra of uncured and cured SDBSs were obtained from the region between 4000 and 650 cm^1^, with 32 scans at 4 cm^1^.

Using FTIR software (IRsolution), a graphic was obtained by associating absorbance peaks with monomer functional groups: aliphatic carbon double-bond absorbance peak intensity (at 1638 cm^1^) and that of the aromatic component (at 1608 cm^1^; reference peak). After obtaining the absorbance values (R cured and R uncured), DC was calculated using Equation 1.

Equation 1: Formula to calculate DC

$$DP=\left(1-\frac{R\;cured}{R\;uncured}\right)\times \;100$$

### WS/WSB tests

The specimens were stored in desiccators at 37°C, in buckets containing silica gel (Synth, Blue Mesh 2-4 mm, São Paulo, SP, Brazil). They were weighed daily on an analytical balance (GR-202, A & D Engineering, Inc., San José, California, USA) with 0.01 mg legibility to obtain a constant mass value (M1) without water loss (oscillation 0.0002 g). Subsequently, the samples were stored in distilled water at 37°C for approximately 10 days. Before weighing, each specimen was carefully dried with a paper towel. When constant weight was obtained, this value was recorded as M2. After this second weighing, the specimens were subjected to an ≈10-day drying process, in which new weights (M3) were obtained, observing the limit of 0.0002 g^6,15,18^. The WS/WSB values in micrograms *per* cubic millimeter (μg/mm^3^) were calculated using the following equations:

### Statistical analysis

Data were collected, and the normal distribution and homogeneity of the variances were assessed respectively by Kolmogorov–Smirnov and Levene’s tests. For DC and WS/WSB tests, data were submitted to two-way analysis of variance, followed by Tukey’s test for individual comparisons (p<0.05). Statistical analysis was performed with the software Statistica 10.0 (StatSoft Inc., Tulsa, Oklahoma, USA).

## Results

### DC

Laser and SDBSs were significant factors (p<0.0001). When associated with laser, both SDBSs presented higher values (p<0.0001). Single Bond (SB) demonstrated a higher DC than Single Bond Universal (SU) (p<0.0001). Additionally, the interaction between both factors was statistically significant (p=0.00007).

### WS/WSB

In these analyses, only the SDBS was a significant factor (WS/p<0.0001 and WSB/p=0.000002). Higher values were obtained by SB. Laser was not significant and significant for WS and WSB, respectively (WS/p=0.510 and WSB/p=0.271).

## Discussion

Preheating was performed before curing the resin-based dental materials. Heating these materials favors the increase of radical mobility^7^, promoting higher DC and lower WS/WSB1m^1,4,25^. Therefore, laser irradiation has also been indicated to heat the adhesive system and improve these properties.

The first null hypothesis tested in this study was rejected, as laser irradiation provided higher DC for all SDBSs (Table 1). This performance is attributed to the solvent evaporation promoted by the increase in temperature. This hypothesis was shown by Batista, et al.^2^ (2015), using an Nd:YAG laser. Vale, et al.^25^ (2014) assessed the DC and WS/WSB by preheating (60°C for 2 hours) a single-bottle adhesive system, and observed their improvement. However, as this study was performed in laboratory, the high temperature was not considered to create pulp damage. In clinical use, the temperature would limit its indication. As the laser diode promoted a variation in temperature of approximately 6°C, varying from 20.98 to 27.21°C for these SDBSs during their application (unpublished data), laser diodes can be more advantageous regarding biological conditions as well.

**Table 1:** Mean (%) and standard deviations for the tested groups by degree of conversion

SDBS	Control	Laser diode
SB	73.00±0.39^Aa^	87.00±0.13^Bc^
SU	71.50±1.75^Ab^	78.00±1.96^Bd^

N=5, p<0.05

Uppercase letters represent comparisons between columns for each test

Lowercase letters represent comparisons between rows for each test

Another rationale that supports the improvement of the DC and WS/WSB of dental adhesives is related to the effect of air-drying on solvent evaporation. Bail, et al.^1^ (2012) observed that the air-drying heated at 40°C for a period of 15-60 s could promote higher DC and lower WS/WSB, which could be a simple strategy. These authors claimed that this alternative increases for a long time the kinetic energy of the molecules in adhesive systems, promoting greater vibration, thereby helping break intermolecular bonds between the solvent and polar groups of the resin comonomers. It promotes solvent evaporation and optimizes the DC. Moreover, the increase in temperature also increases vapor pressure, improving its evaporation. However, oxygen can inhibit the polymerization of resin-based material, which was not considered in this study^8,15^.

Based on the literature, the performance of the laser diode on the SDBSs suggests that this could be an interesting option, as it favors the improvement of the DC in safe and more realistic clinical conditions. Batista, et al.^2^ (2015) reported that the use of an Nd:YAG laser on the uncured adhesive promoted a greater degree of evaporation of solvents, and this was directly influenced by their physicochemical properties. As the tested bonding systems contain solvents, the use of laser could promote their evaporation simultaneously with the improvement of cross-link reactions, which may be responsible for the greater DC.

However, when the SDBSs were compared, SB performed better than SU. This can be partially attributed to the presence of 10-methacryloyloxydecyl dihydrogen phosphate (MDP) and a polyalkenoic acid copolymer in SU^5,20^. MDP was introduced as a functional acid monomer that must interact with dentin for better performance. Once applied, the polyalkenoic acid copolymer may compete for calcium-bonding sites with the MDP monomer and, due to its high molecular weight, could prevent the conversion of monomers during polymerization^20,26^. As the DC was assessed without the influence of dentin, the conversion of this monomer was likely reduced due to the impossibility of the interaction with dentin.

Therefore, the heating advantages of laser in relation to other investigated heat treatments are that, in addition to having the ability of helping solvent evaporation, some authors report that laser irradiation can also promote “the development of a new substrate, in which dentin substrate and adhesive would be fused by laser action, raising bond strength values^19^.

The second null hypothesis tested in this study was accepted (Table 2); laser diode did not affect the WS/WSB of SDBSs. It is possible that the heat of SDBSs by laser irradiation with 0.8 W of power was not enough to help breaking the intermolecular bonds between the solvent and the polar groups of the SDBS. Despite the differences in technique proposed by the studies of Maenosono, et al.^17^ (2015) and Gonçalves, et al.^13^ (1999) (type of laser, irradiation time, area, and application mode), both studies show positive results, with increased bond strength values. Therefore, it is important to emphasize that the increase in temperature on the subsurface experienced during laser irradiation of dentin bonding systems, and the consequent solvent evaporation, are strongly dependent on irradiation parameters, and that further studies are required in this area.

**Table 2:** Mean (μg/mm3) and standard deviations for the tested groups by water sorption and solubility

SDBS	Control	Laser diode	Control	Laser diode
SB	208.59±6.38^Aa^	214.48±10.37^Aa^	86.70±6.21^Aa^	88.73±7.27^Aa^
SU	121.04±6.88^Ab^	125.76±8.97^Ab^	78.20±4.75^Ab^	76.08±4.85^Ab^

N=10, p<0.05

Uppercase letters represent comparisons between columns for each test

Lowercase letters represent comparisons between rows for each test

Silva, et al. ^22^ (2016) observed significantly reduced variation of intrapulpal temperature and microtensile bond strength to dentin when submitted to an adhesive technique using laser irradiation associated with simulated pulpal pressure, and the authors related the presence of liquids within the pulp chamber to the altered absorption of heat generated by laser energy. Therefore, it is important to consider the amount of adhesive in clinical situations that is exposed to water coming from the pulp. This water could interfere in this process by impeding evaporation of the solvent due to molecular weight and vapor pressure, or the water could be removed during the laser irradiation.

In this study, SB showed higher WS and WSB compared to SU. The compositions of these systems differ, essentially due to the presence of MDP in SU. Most likely, it contributed to providing better resistance in a moist environment, as it is a functional acidic monomer less prone to hydrolytic degradation than BisGMA^19^. According to Daronch, Rueggeberg, and De Goes^7^(2005), heating reduces material viscosity and increases the mobility of the radicals and reacted monomers, resulting in further curing and higher DC.

The results obtained in this investigation could explain the findings of Maenosono, et al.^17^(2015), who also employed the use of laser diode with SDBSs. Groups treated with laser showed better performance regarding bonding strength^13^. Furthermore, the results may also explain the findings of Franke, et al*.* ^11^(2006), Ghiggi, et al.^12^(2010), and Marimoto, et al.^19^(2013). In these studies, the authors employed the same Nd:YAG laser (with different parameters) with the same purpose. The laser diode seems to be more attractive due to its proximity wavelength, versatility, smaller dimensions, and lower cost^17^.

It can be speculated that higher DC values could increase the immediate bond strength, with improved mechanical properties^10,16^ in the “newly formed substrate.” It is observed that laser irradiation on SDBSs looks promising and may become a potential clinical resource. Further studies are necessary to provide a more appropriate protocol to improve the mechanical properties of SDBSs.

## Conclusion

Considering the limitations of this study, we can conclude that laser diode irradiation improved the DC of the tested SDBSs, with no impact on WS and WSB.
